# Temporal expression and cellular origin of CC chemokine receptors CCR1, CCR2 and CCR5 in the central nervous system: insight into mechanisms of MOG-induced EAE

**DOI:** 10.1186/1742-2094-4-14

**Published:** 2007-05-07

**Authors:** Sana Eltayeb, Anna-Lena Berg, Hans Lassmann, Erik Wallström, Maria Nilsson, Tomas Olsson, Anders Ericsson-Dahlstrand, Dan Sunnemark

**Affiliations:** 1 Department of Clinical Neuroscience, Center for Molecular Medicine, Neuroimmunology Unit, Karolinska Institute, S-171 76 Stockholm, Sweden; 2 Department of Pathology, Safety Assessment, AstraZeneca R&D Södertälje, S-15185 Södertälje, Sweden; 3 Brain Research Institute, University of Vienna, Vienna, Austria; 4 Department of Disease Biology, Local Discovery Research Area CNS and Pain Control, AstraZeneca R&D Södertälje, S-151 85 Södertälje, Sweden

## Abstract

**Background:**

The CC chemokine receptors CCR1, CCR2 and CCR5 are critical for the recruitment of mononuclear phagocytes to the central nervous system (CNS) in multiple sclerosis (MS) and other neuroinflammatory diseases. Mononuclear phagocytes are effector cells capable of phagocytosing myelin and damaging axons. In this study, we characterize the regional, temporal and cellular expression of CCR1, CCR2 and CCR5 mRNA in the spinal cord of rats with myelin oligodendrocyte glycoprotein-induced experimental autoimmune encephalomyelitis (MOG-EAE). While resembling human MS, this animal model allows unique access to CNS-tissue from various time-points of relapsing neuroinflammation and from various lesional stages: early active, late active, and inactive completely demyelinated lesions.

**Methods:**

The expression of CCR1, CCR2 and CCR5 mRNA was studied with *in situ *hybridization using radio labelled cRNA probes in combination with immunohistochemical staining for phenotypic cell markers. Spinal cord sections from healthy rats and rats with MOG-EAE (acute phase, remission phase, relapse phase) were analysed. In defined lesion stages, the number of cells expressing CCR1, CCR2 and CCR5 mRNA was determined. Data were statistically analysed by the nonparametric Mann-Whitney U test.

**Results:**

In MOG-EAE rats, extensive up-regulation of CCR1 and CCR5 mRNA, and moderate up-regulation of CCR2 mRNA, was found in the spinal cord during episodes of active inflammation and demyelination. Double staining with phenotypic cell markers identified the chemokine receptor mRNA-expressing cells as macrophages/microglia. Expression of all three receptors was substantially reduced during clinical remission, coinciding with diminished inflammation and demyelination in the spinal cord. Healthy control rats did not show any detectable expression of CCR1, CCR2 or CCR5 mRNA in the spinal cord.

**Conclusion:**

Our results demonstrate that the acute and chronic-relapsing phases of MOG-EAE are associated with distinct expression of CCR1, CCR2, and CCR5 mRNA by cells of the macrophage/microglia lineage within the CNS lesions. These data support the notion that CCR1, CCR2 and CCR5 mediate recruitment of both infiltrating macrophages and resident microglia to sites of CNS inflammation. Detailed knowledge of expression patterns is crucial for the understanding of therapeutic modulation and the validation of CCR1, CCR2 and CCR5 as feasible targets for therapeutic intervention in MS.

## Background

Multiple sclerosis (MS) is the most common non-traumatic cause of neurological disability in young adults in the Western world. It is a chronic inflammatory disease, characterized by the appearance of focal demyelinated plaques within the central nervous system (CNS) [1]. Essential aspects of MS lesions are mimicked in models of experimental autoimmune encephalomyelitis (EAE), and thus autoimmunity is considered an important pathogenetic factor in the disease [2].

It is generally assumed that inflammation caused by the penetration of circulating leukocytes through the blood brain barrier, drives demyelination and axonal injury within the lesions [3]. Different patterns of demyelination have been described in early active MS lesions, suggesting discrete pathways that may lead to the common endpoint of myelin injury [4,5]. Although the pathogenetic mechanisms leading to demyelination and tissue injury are not fully understood, activated macrophages and microglia seem to play a central role in the destructive process both in MS and in EAE [6,7]. In accordance with this assumption, elimination of macrophages or microglia has been shown to suppress clinical and histopathological manifestations in rodent models for MS [8,9].

Chemokines stimulate migration of inflammatory cells towards tissue sites of inflammation by establishing a chemotactic gradient that attracts specific subsets of leukocytes [10,11], and there appears to be organ-specific molecular details for leukocyte trafficking [12]. Chemokines act as ligands on a subgroup of G-protein coupled seven transmembrane domain receptors called chemokine receptors [13,14]. Leukocytes expressing a variety of inflammatory chemokine receptors, most consistently CCR1, CCR2, and CCR5, have been identified in diverse inflammatory tissues and fluids, including synovial fluid from rheumatoid arthritis patients [15], joints of arthritic mice [16], MS brain lesions [17-19] and in neurological disease models including EAE [20,11,23].

Even though the chemokine network is notorious for its redundancy and receptor promiscuity in vitro, studies in rodent models for MS have utilized techniques for genomic deletion of chemokines [24], chemokine receptor genes [22,25,26], function-blocking antibodies [27] or receptor antagonists [28,29], to demonstrate a non-redundant role for individual chemokine receptors and their ligands.

Here we present data from a series of experiments which was designed to characterize the expression of CC chemokine receptors CCR1, CCR2 and CCR5 in the spinal cord of rats with experimentally induced MS-like disease, myelin oligodendrocyte glycoprotein-induced EAE (MOG-EAE) [30]. These receptors were selected for analysis as they have previously been demonstrated to control migration of macrophages into inflammatory foci. The model employed in this study typically exhibits a primary progressive or relapsing-remitting disease course that in many aspects mimics MS, with the formation of focal areas of demyelination [31] and axonal injury and loss [32].

Our results demonstrate a prominent accumulation of monocytes and macrophages expressing CCR1, CCR2 or CCR5 mRNA within and around inflammatory foci in the spinal cord of rats with EAE, thus identifying potential determinants for trafficking of these cells to the CNS. These findings are discussed in relation to therapeutic strategies to interfere with macrophage-mediated demyelination and axonal injury in MS [33].

## Methods

### Animals

Female DA.RT1av1 rats at 10 to 14 weeks of age (150–200 g) were obtained from B&K Universal AB (Sollentuna, Sweden). All rats were housed under specific pathogen-free conditions, caged in groups of four at constant room temperature on a 12-hour light-dark cycle, with food and water freely available to keep the influence of additional environmental factors, besides immunization as low as possible. All animal experiments were approved and performed in accordance with Swedish national guidelines.

### Preparation of MOG

Recombinant rat MOG corresponding to the N-terminus of the protein (amino acids 1–125) was expressed in E. coli and purified to homogeneity by chelate chromatography as previously described [34]. The purified protein in 6 M urea was dialyzed against PBS to obtain a preparation that was stored at -20°C.

### Induction and assessment of EAE

Rats were anaesthetized with isoflurane (Baxter Medical AB, Kista, Sweden) and injected subcutaneously at the base of the tail with 0.2 ml inoculum, containing 20 μg recombinant rat MOG (amino acids 1–125) in saline, emulsified (1:1) with Incomplete Freund's adjuvant (IFA) (Difco, Detroit, MI) [31]. Rats were clinically scored and weighed daily from day 7 after immunization until day 29 after immunization by two alternating investigators. The clinical scoring was as follows: 0 = no illness, 1 = tail weakness or tail paralysis, 2 = hind leg paraparesis, 3 = hind leg paralysis, 4 = complete paralysis, moribund state, or death. A disease remission was defined as an improvement in disease score from either 3 or 4 to 1, or from 2, 3, or 4 to 0 that was maintained for at least 2 days consecutively. A relapse was defined as an increase in the clinical deficit of at least 2 points that lasted for at least 2 days or more. Healthy rats served as controls. At various time points after immunization (day 8–29) rats were killed with CO_2 _and perfused via the ascending aorta with sterile PBS and 4% paraformaldehyde. The spinal cords were quickly dissected out and routinely embedded in paraffin wax until use.

### Histopathology

**Figure 1:**
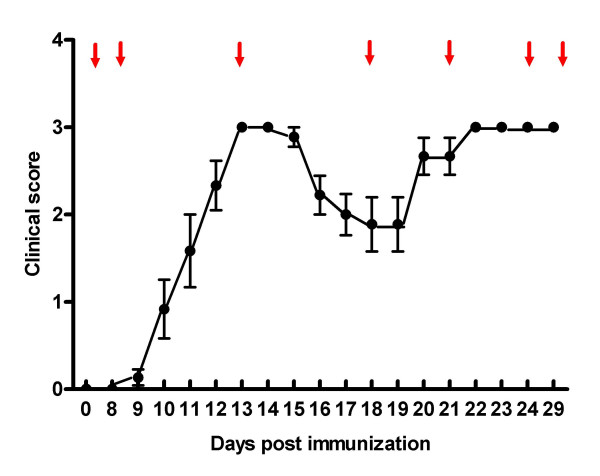
**Sampling of rats from various clinical stages of MOG-EAE**. Mean clinical score in female DA rats (*n *= 30), evaluated daily 8–29 days after immunization with 20 μg recombinant rat MOG in incomplete Freund's adjuvant. The arrows indicate selected time-points at which subsequent kinetic analyses were performed. Rats (*n *= 3/time-point) which conformed in the clinical score curve were chosen for histopathology and evaluation of CCR1, CCR2 and CCR5 mRNA expression in the spinal cord. Vertical bars represent mean and standard error of the mean.

Histopathological evaluation was performed on paraformaldehyde-fixed, paraffin-embedded sections of the spinal cord sampled at day zero, 8, 13, 18, 21, 24, and day 29 after immunization (Figure 1). Serial 4 μm thick paraffin sections were cut on a microtome and stained with haematoxylin and eosin (H&E), Luxol fast blue (LFB)/periodic acid Schiff'(PAS) and Bielschowsky silver impregnation to assess inflammation, demyelination, and axonal loss, respectively [31].

### Preparation of radioactively labelled cRNA probes

Preparation of radioactively labelled cRNA probes encoding the CCR1, CCR2 and CCR5 receptors was carried out as previously described [35]. Briefly, the CCR1, CCR2 and CCR5 cRNA probes were transcribed from cDNA fragments cloned into pBluescript SKII plasmid vector (Stratagene, La Jolla, CA). These cDNA fragments correspond to bases (a 1280 bp cDNA fragment encoding part of rat CCR1, accession number U92803; (a 1000 bp cDNA fragment encoding part of rat CCR5 accession number U77350); (a 310 bp cDNA fragment encoding part of rat CCR2, accession number U92803) and were generated by RT-PCR using sequence-specific oligonucleotide primers. The identity of the cloned cDNA fragments was finally confirmed by sequencing and database comparisons. Restriction enzymes and RNA polymerases were obtained from Promega (Madison, WI). Antisense and sense cRNA probes were transcribed in vitro with T3 or T7 RNA polymerase in the presence of ^35^S-uridine triphosphate (^35^S-UTP; NEN-DuMedical, Sollentuna, Sweden). After removal of unincorporated nucleotides by Quick Spin columns (Boehringer Mannheim, Indianapolis, IN), the specific activities of all the probes were 1–3 × 10^9 ^dpm/ug. As controls, radio labelled probes were transcribed in the sense orientation and hybridized to slides as processed in parallel.

### In situ hybridization histochemistry

To detect expression of CCR1, CCR2 and CCR5 mRNA, in situ hybridization experiments were performed on paraffin-embedded tissue sections from rat spinal cord sampled at day zero, 8, 13, 18, 21, 24, and day 29 after immunization (Figure 1). Hybridization and autoradiography were carried out according to protocols previously described by Swanson et al [35], although post-fixation and treatment with acetic anhydride and proteinase K were replaced with an antigen retrieval technique. Briefly, spinal cord sections were mounted on Superfrost plus slides (Super Frost Plus, Pittsburgh, USA) and dried under vacuum overnight after defatting in xylene, pre-treated in a microwave oven at approximately 97°C in 10 mM SSC (pH 6.0) for 10 min and dehydrated in ethanol. As controls, radio labelled sense probes were hybridized to slides processed in parallel. After application of 100 ul of hybridization solution containing 10^6 ^cpm of the cRNA probes, the slides were cover-slipped and incubated at 60°C for 16 to 20 hours. Slides were subsequently washed in 4 × SSC, pH 7.0, digested in 20 μg/ml ribonuclease A solution at 37°C for 30 minutes, washed in decreasing concentrations of SSC, ending with 0.1 × SSC for 30 minutes at 70°C, dehydrated with ethanol, and dried.

### Immunohistochemistry

To identify the cellular phenotypes of the CCR1, CCR2 and CCR5 expressing cells, immediately following the high stringency post hybridization washes (0.1 × SSC at 75°C) immunohistochemistry was performed with a panel of cell-specific markers. Slides were pre-treated using an antigen retrieval technique (5 × 5 min boiling in 10 mM Na-citrate buffer, pH 6.0 at 97°C in a microwave oven). The following monoclonal primary antibodies were used: an antibody specific for rat T cells (W3/13, Harlan Sera Lab), an antibody specific for phagocytic rat monocytes and macrophages (ED-1, Serotec), and an antibody reactive with glial fibrillary acidic protein (GFAP) for the identification of astrocytes (clone G-A-5, Boehringer Mannheim). The primary antibodies were diluted 1/30 (W3/13), 1/500 (ED-1) and 1/20 (G-A-5). A biotinylated sheep anti-mouse antibody (Life Sciences) served as the secondary reagent, with the avidin biotin peroxidase (ABC) detection system (ABC Elite, Vector Laboratories) and diaminobenzidine as chromogen. Finally, a biotinylated lectin (GSA/B4, Vector Laboratories) combined with the ABC detection system was used for the detection of macrophages and microglia in various stages of activation. Control sections were incubated without primary antibody as control of specificity of the staining. Slides were exposed to a phosphorimager screen (Fujifilm, Sweden), followed by exposure to X-ray film (Beta max, Kodak) and finally coated with autoradiographic photo emulsion (NTB2, Kodak). After 14–28 days exposure to emulsion at 4°C the slides were developed in Kodak D-19 developer for 4 minutes at 17°C. Slides were then counterstained with hematoxylin and coverslipped.

### Selection of demyelinated plaques and definition of lesion stages

In a total of 11 spinal cord sections from 4 rats in the relapse stage (days 21–29 pi.) and 1 rat in the acute stage (day 13 pi.), 17 lesions (plaques) were selected and defined according to the state of inflammatory activity and demyelination as described by Brück et al [36]. Early active (EA) lesions were characterized by dense infiltrates of macrophages, lymphocytes and microglia. Myelin sheaths were in the process of disintegration and macrophages contained LFB-stained myelin degradation products. Late active (LA) lesions were still densely populated by macrophages. Damaged myelin had been removed from the axons and macrophages contained PAS-positive myelin degradation products. Inactive and demyelinated (IADM) lesions showed no evidence of ongoing tissue destruction at the borders of the plaque. Inflammatory cells were present, although at lower density than in EA and LA lesions. Macrophages in IADM lesions did not display LFB or PAS staining. The region in the immediate vicinity of lesions, showing no microscopical signs of demyelination, was defined as periplaque white matter (PPWM). Four out of 17 lesions were defined as EA, 7 as LA and 6 as IADM. Seven PPWM areas were included for comparison.

### Morphometry

Spinal cord sections were photographed with a Kappa DX-20 digital camera mounted on a Nikon E600 microscope. In each of the defined lesion areas, the number of CCR1, CCR2 and CCR5 mRNA-expressing cells was determined in 1–2 standardized microscopic fields (1.9 × 10^4 ^μm^2^) using the Analysis Pro system (Euromed Networks, Stockholm, Sweden). In a few cases, the number of cells was manually counted. In total, 33 fields of 1.9 × 10^4 ^μm^2 ^each were included in the morphometric analysis.

### Statistics

The nonparametric Mann-Whitney U test was used for analysis of the morphometric data. A *p *value < 0.05 was considered to be statistically significant.

## Results

### Study design

The DA.RT1av1 rat strain develops MS-like disease with a relapsing-remitting clinical disease course when immunized with MOG [37,31]. Onset of disease is clinically observable 9 to 13 days after immunization (Fig. 1). At the histopathological level, MOG-EAE mimics many features of human MS, thus being considered as one of the best experimental models of choice for preclinical studies aimed at elucidating the mechanistic basis of MS [31].

A key issue in understanding the pathogenesis of MS is the reliable identification of phagocytes capable of degrading myelin. Since infiltration of leukocytes including monocyte-derived macrophages into the CNS is a key step in the pathogenesis of MS [38], we designed this study to identify chemokine receptors that may control infiltration of monocyte-derived macrophages into inflammatory CNS lesions of rats with MOG-EAE. CCR1, CCR2 and CCR5 have all been previously demonstrated to control migration of macrophages into inflammatory foci.

Tissue sections sampled at regular intervals throughout the spinal cord were collected from healthy control rats and from representative MOG-EAE rats that were harvested at different stages of their disease development (Fig. 1). This included rats in the pre-symptomatic (day 8), acute (day 13), remission (day 18), as well as rats at various stages of relapse (days 21, 24 and 29) after immunization. The expression of CCR1, CCR2 and CCR5 was assessed at the mRNA level using in situ hybridization with gene-selective ^35^S-labeled anti-sense cRNA probes in combination with immunohistochemical staining for phenotypic cell markers. The expression of CCR1, CCR2 and CCR5 was further studied in relation to a detailed outline of the inflammatory lesions, where each lesion area was characterized according to state of inflammatory activity and demyelination, as previously described by Brück et al [36].

### Distribution of CCR1, CCR2 and CCR5 mRNA in the rat spinal cord

**Figure 2:**
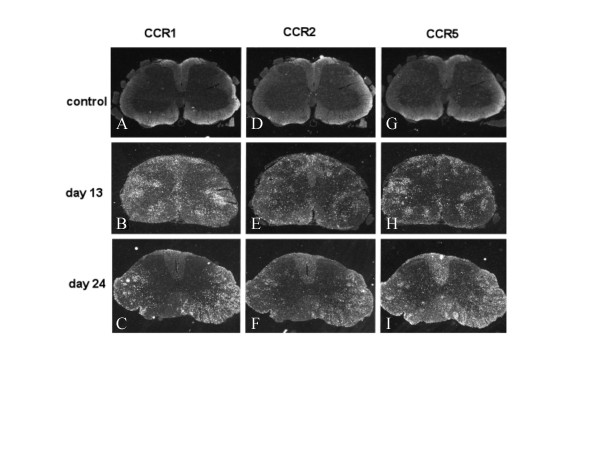
**Distribution of CCR1, CCR2, CCR5 mRNA expressing cells at different time points in the spinal cord of MOG-EAE rats**. In situ hybridization with ^35^S-labelled antisense cRNA probes encoding rat CCR1, CCR2 and CCR5 to coronal sections from the lumbar segment of spinal cord of rats with MOG-EAE. Cells expressing CCR1, CCR2 and CCR5 mRNA are visualized by dark field illumination of the photo emulsion-dipped slides. Intensive signals for CCR1 and CCR5 mRNA, and moderate signals for CCR2 mRNA, were detected on days 13 (B, E, H) and 24 (C, F, I) post immunization. No signal for CCR1, CCR2 or CCR5 mRNA was detected in healthy control animals (A, D, G). No signal was detected in control sections hybridized with sense probe (not shown).

**Figure 3:**
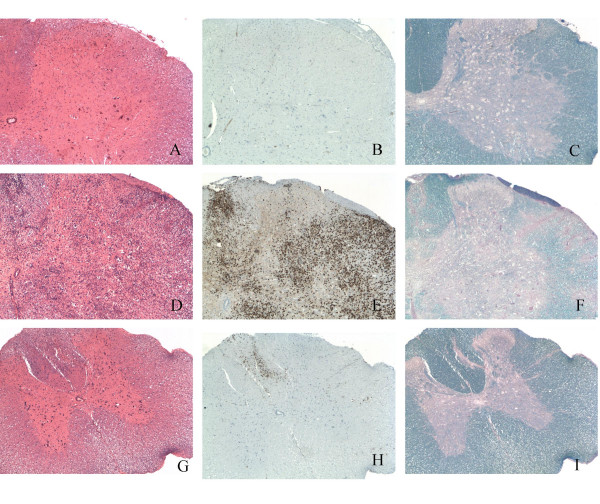
**Histopathological features of MOG-EAE during the acute and remission stages**. Spinal cord sections from a rat in the acute stage (day 13 post immunization) of EAE show extensive inflammation involving the white and grey matter (D), with marked demyelination in the inflammatory areas (F). The majority of the infiltrating inflammatory cells are macrophages, as evidenced by positive staining for the ED-1 marker (E). During the remission phase (day 18 post immunization), inflammation (G) and infiltration of macrophages (H), as well as demyelination (I) are substantially reduced. A normal control rat is included for comparison (A, B, C). H&E staining (A, D, G), ED-1 immunohistochemistry (B, E, H), LFB/PAS staining (C, F, I). Magnification: lens × 4.

No expression of CCR1, CCR2 or CCR5 was detected within the spinal cord of healthy control rats (Fig. 2A, 2D, 2G) or MOG-EAE rats in the pre-symptomatic phase on day 8 p.i. (data not shown). Histopathological evaluation of MOG-EAE rats in the acute phase (day 13) revealed marked inflammatory lesions in the white and grey matter of the spinal cord (Fig. 3D). Within the inflammatory infiltrates, numerous actively phagocytosing macrophages were identified (Fig. 3E) corresponding to areas undergoing demyelination (Fig. 3F). A strong labelling for CCR1 and CCR5 mRNA was observed over cells within the inflammatory and demyelinating areas in rats with acute MOG-EAE (Fig. 2B, 2H). In contrast, only weak to moderate labelling for CCR2 mRNA was detected during the initial acute phase, over cells within a few restricted areas of the spinal cord displaying focal inflammation and demyelination (Fig. 2E).

During the clinical remission phase (day 18), inflammation and demyelination in the spinal cord were considerably diminished (Fig. 3G, 3I) and the number of infiltrating macrophages clearly reduced (Fig. 3H). This coincided with substantially reduced expression of CCR1, CCR2 and CCR5 in the spinal cord (data not shown). Enhanced expression of CCR1, CCR2 and CCR5 mRNA was subsequently observed over cells within inflammatory aggregates during the early stages of the clinical relapse (day 21) and on day 24 p.i. (Fig. 2C, 2F, 2I). At a later phase of the clinical relapse (day 29), a moderate expression of CCR1 mRNA was detected over cells that tended to distribute to sub-areas of the inflammatory aggregates (data not shown). Expression of CCR2 mRNA was substantially reduced, while CCR5 mRNA was strongly expressed in the white matter of the spinal cord. No signal above the general background level could be detected in sections hybridized with CCR1, CCR2 and CCR5 sense cRNA probes (data not shown).

**Figure 4:**
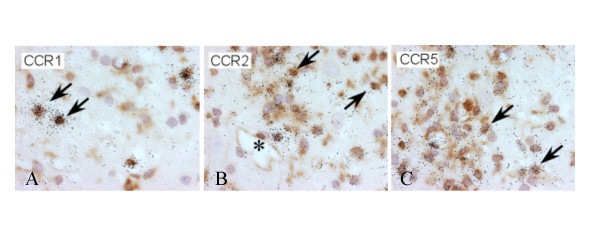
**Cellular phenotype of chemokine receptor mRNA expressing cells in MOG-EAE**. High magnification bright-field photomicrographs of spinal cord sections from MOG-EAE rats processed for combined GSA/B4 immunohistochemistry and CCR1, CCR2, CCR5 mRNA in situ hybridization. Cells expressing CCR1 (A), CCR2 (B) or CCR5 (C) mRNA are positively stained with GSA/B4, identifying them as macrophages/microglia.

To determine the identity of the CC receptor-expressing cells we subsequently employed a combination of in situ hybridization and immunohistochemistry, using markers for infiltrating monocytes, resident macrophages and microglia (lectin GSA/B4; labels all macrophages and microglia), actively phagocytosing cells (antibody against ED1; recognizes a lysosomal membrane antigen in actively phagocytosing cells), T-cells (W3/13) and astrocytes (GFAP). Expression of CCR1, CCR2 and CCR5 mRNA was detected exclusively in ED-1+ cells and in the amoeboid form of the GSA/B4+ cells, indicating that these chemokine receptors are expressed by cells of the macrophage/microglia lineage, but not by T cells or astrocytes (Fig. 4A, 4B, 4C).

### Quantification of CCR1, CCR2 and CCR5 mRNA-expressing cells in relation to the stage of demyelinating activity

**Figure 5:**
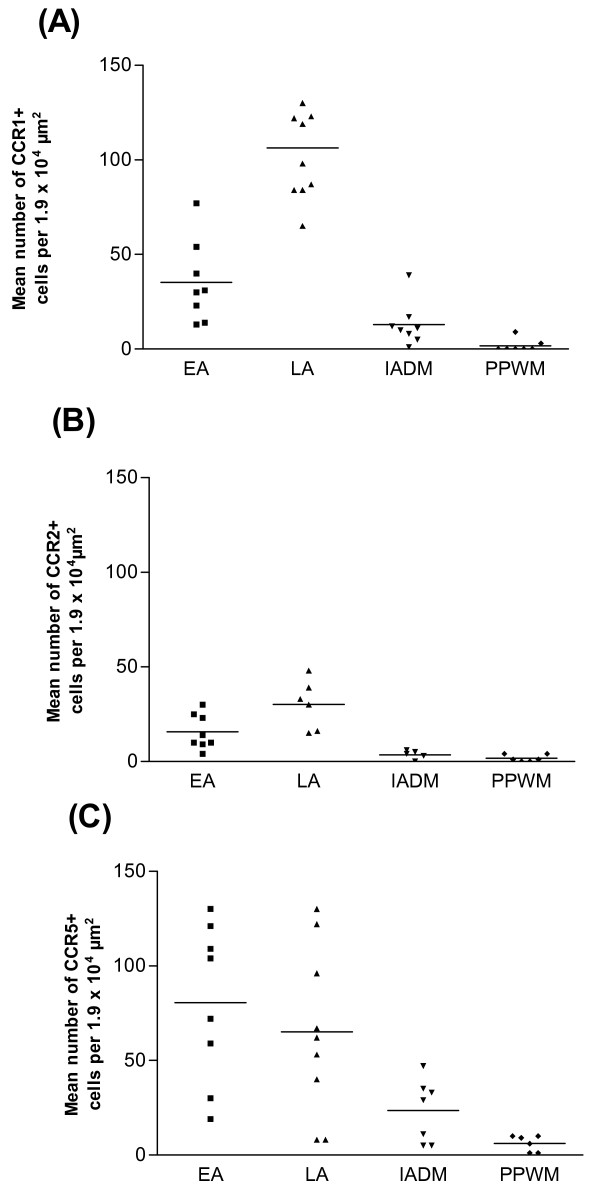
**Quantification of CCR1, CCR2 and CCR5 mRNA expressing cells in defined lesional stages**. Mean numbers of CCR1+ cells (A), CCR2+ cells (B) and CCR5+ cells (C) per square unit in spinal cord sections from MOG-EAE rats. Lesions were characterized as EA = early active, LA = late active and IADM = inactive demyelinated. PPWM = periplaque white matter. Bar = mean.

The sampling at specific time points was complemented by detailed lesion maps where each lesion area was characterized for its state of inflammatory activity and demyelination/remyelination as previously described by Brück et al [36]. A detailed analysis of CCR1, CCR2 and CCR5 in EAE rats revealed dynamic changes in their relative expression within those sub-areas in the spinal cord. Areas directly adjacent to the inflammatory lesions (the PPWM areas) contained a low but detectable number of chemokine receptor-expressing cells, with CCR5+ cells being detected at somewhat higher abundance (Table 1, Fig. 5A–C). The active border zone of the inflammatory lesions, the so called EA (early active) lesions where the inflammatory and demyelinating activity is most intensively manifested, exhibited sharply elevated numbers of cells expressing CCR1 (P < 0.001 vs PPWM), CCR2 (P < 0.05 vs PPWM) or CCR5 (P < 0.001 vs PPWM) mRNA, with the relative proportions of CCR5 > CCR1 > CCR2 (Table 1, Fig. 5A–C).

**Table 1:** Numbers of CCR1, CCR2 and CCR5 mRNA-expressing cells per square unit (1.9 × 10^4 ^μm^2^) in rat EAE lesions (mean ± SEM)

	**CCR1**	**CCR2**	**CCR5**
**PPWM**	1.7 ± 1.3	1.7 ± 0.8	6.2 ± 1.7
**EA**	35.3 ± 7.6^a^	15.6 ± 3.3^a^	80.5 ± 14.8^a^
**LA**	106.4 ± 8.5^b^	30.2 ± 5.3^b^	65.1 ± 14.8^a^
**IADM**	12.9 ± 4.1^c^	3.6 ± 1.0	23.6 ± 6.3

^a ^Statistically significant against IADM lesions and PPWM areas

^b ^Statistically significant against EA and IADM lesions and PPWM areas

^c ^Statistically significant against PPWM areas

EA = early active lesions, LA = late active lesions, IADM = inactive completely demyelinated lesions, PPWM = periplaque white matter.

In inflammatory spinal cord lesion areas representing later, but still active, stages of demyelination (LA or late active lesions), CCR1 (*P *< 0.0001) and CCR2 (*P *< 0.05) expressing cells aggregated at increasing numbers as compared to the EA lesions, whereas the CCR5+ cells were slightly reduced in numbers as compared to the EA lesion areas (Table 1, Fig. 5A–C). The relative proportions of chemokine receptor expressing cells within the LA areas were CCR1 > CCR5 > CCR2. In comparison with LA areas, there was a sharp decline in the number of cells expressing CCR1 (*P *< 0.0001), CCR2 (*P *< 0.05) and CCR5 (*P *< 0.05) within the so called IADM (inactive and demyelinated) lesions areas characterized by complete demyelination and low inflammatory and demyelinating activity. In these areas the majority of the chemokine receptor expressing cells were CCR5+ cells, whereas the CCR2+ cells were most infrequently detected.

## Discussion

Mononuclear phagocytes are central components of brain lesions in MS and are believed to be effector cells causing demyelination and axonal injury in MS [38]. The current study was carried out to further identify chemokine receptors that may control infiltration of monocyte-derived macrophages into inflammatory CNS lesions of rats with MOG-EAE, a widely used chronic model for MS. The expression of chemokine receptors CCR1, CCR2 and CCR5 was studied in spinal cord tissues from healthy control and MOG-EAE rats sampled at the preclinical, acute, remission and relapse phases of the disease. The CNS lesions were defined according to previously described criteria for MS [36], thus enabling a direct comparison between our chronic rat model and MS.

Our results demonstrate that the acute phase of MOG-EAE was associated with distinct expression of CCR1, CCR2, and CCR5 by cells of the macrophage/microglia lineage within the CNS lesions. CCR1 and its ligands CCL3, CCL5 and CCL7 have previously been shown to be expressed within inflammatory brain lesions in MS [18,39-41], and CCL3 has been demonstrated in cerebrospinal fluid of MS patients with relapsing-remitting disease course [42]. In MS lesions, CCR1 expression, at the protein level, has been associated with the early stage of monocyte infiltration into the CNS, and with the active demyelinating border zone of lesion, while in inactive areas of lesions, where myelin phagocytosis is completed, only a minority of macrophages expresses CCR1 [7].

Interestingly and consistent with the situation in MS, we have found here a similar distribution pattern of CCR1 mRNA in our rat model, with an increased expression on ED-1 and GSI-B4 isolectin-labelled cells in early active (EA) and late active (LA) demyelinating lesions. During the remission phase of the disease, CCR1 mRNA expression was substantially reduced. This reduction in CCR1 mRNA expression coincided with diminished inflammation and demyelination, and with considerably reduced numbers of infiltrating macrophages.

These data confirm previous findings from our laboratory showing CCR1 mRNA to be preferentially expressed by macrophages in areas of active demyelination, while resting microglia within the spinal cord of control and in rats with MOG-induced EAE are uniformly negative for CCR1 mRNA and protein [43]. The importance of CCR1 in the pathogenesis of EAE is emphasized by the fact that immunoneutralization of CCL3 [44], DNA vaccination [45], or genomic deletion of the CCR1 gene [22], reduces clinical disease. Taken together, the results of the present study and from previous ones on the role of CCR1 and its ligand CCL3 in the pathogenesis of MS [39,40,42] and EAE [22,44,46], have provided evidence for an important role of CCR1 in MS and EAE.

Moreover, our group has previously shown that a low-molecular weight CCR1 selective antagonist reduces infiltration of leukocytes into the CNS, as well as demyelinating activity, axonal pathology, and paralysis, during the effector stage of the disease [47]. Thus, administration of a CCR1 selective antagonist alone was sufficient to inhibit the acute paralytic disease in MOG-EAE, suggesting that CCR1 is non-redundant at this early stage of the disease and may provide a feasible target for therapeutic intervention in MS. However, recent clinical trials with a low-molecular weight CCR1 antagonist failed to demonstrate efficacy in patients with relapsing/remitting MS [48-51]. Thus, we propose that CCR1 is a major player in controlling the early proinflammatory events in EAE, and probably in MS, but may be less critical when the demyelination progresses in already established lesions. Many of the discrepancies in results obtained from EAE and MS studies may reflect the fact that EAE experiments are designed to study the induction phase of disease, whereas MS is studied after disease induction, as its cause is unknown [52], and most MS patients do not develop symptoms until inflammation and tissue injury within the CNS have become more established.

We have also demonstrated that CCR2 mRNA is present within spinal cord lesions of EAE rats primarily representing EA and LA demyelinating activity. The co-labelling for isolectin and the marker for phagocytosis, ED-1, as well as their amoeboid morphology, identified those cells as infiltrating macrophages or amoeboid microglia. Our findings confirm previous studies describing the expression of CCR2 and its ligand CCL2 within inflamed brain lesions of rodents with EAE [53], and are in agreement with previous studies demonstrating an important role for CCR2 and CCL2 in controlling infiltration of monocytes to sites of inflammation during relapsing EAE [21].

No significant difference between MS patients and non-inflammatory controls were found in some studies regarding CCR2 expression on monocytes or T cells [54,55], while in other studies expression of CCR2 on circulating monocytes was demonstrated during MS relapse [56]. Moreover, in vivo treatment with IFN-β caused increased expression of CCR2 in MS patients compared to controls [57]. However, the significance of CCL2 and CCR2 in MS is enigmatic, because CCL2 levels are consistently decreased in the CSF of patients with this disease and other chronic neuroinflammatory conditions, despite abundant expression within lesional MS tissues [58]. These interpretations are limited, however, by insufficient knowledge and paucity of studies concerning distribution of CCR2 in MS, due to technical reasons such as restricted availability of commercial antibodies, despite the nonredundant role of CCR2 that demonstrated by using animal models.

Immunoneutralization of CCL2 [21], and genomic deletions of CCR2 [23,25,26], or CCL2 [59] result in a decreased susceptibility to EAE and reduced mononuclear cell infiltration. In a recent study [29], Brodmerckel et al demonstrated a dose-dependent inhibition of macrophage influx in rodent models for EAE and arthritis, following treatment with a selective small molecule CCR2 antagonist. The antagonist was also effective in reducing clinical disease. In the present study, the lower level of expression of CCR2 on infiltrating macrophages in EAE lesions as compared to CCR1 and CCR5, as well as the recent demonstration that CCR2 expressing cells are infrequent in MS lesions [59], may be explained by data from a recent study by Mahad et al [58,60], who used an in vitro model of the blood-brain barrier to demonstrate that T cells and monocytes rapidly down-regulate CCR2 while transmigrating across the barrier in response to presented CCL2. This may possibly be extended to a reduced expression of the receptor even at the mRNA level, and ligand-induced receptor internalization is a well-documented phenomenon among chemokine receptors [61].

CCR5 mRNA was primarily expressed on ED-1 and GSI-B4 isolectin-labelled cells within EA and LA lesions in the spinal cord, with fewer numbers being detected in completely inactive demyelinated (IADM) lesions. Immunohistochemical and morphological characterization identified these cells as infiltrating macrophages and reactive microglia. In line with our findings, monocyte-derived macrophages characterize brain lesions in MS [38] and the abundant expression of a variety of chemokine receptors by cells of monocyte/macrophage lineage is suggestive of a redundancy in the chemokine-mediated control of macrophage function [62]. Most leukocytes found in MS lesions are macrophages, derived either from monocytes or microglia [63]. Despite different origins (ie, resident microglia *versus *hematogenous monocytes), most phagocytic macrophages in MS were shown to express CCR5 within demyelinated lesions [64], and its expression on resident microglial cells and haematogenous monocytes increased during MS lesion evolution [7], confirming our findings here.

In line with this, Mahad et al [40] have previously reported that CCR1 and CCR5 expression in MS lesions differs depending upon the pattern of demyelination and injury. In pattern II lesions, the number of cells expressing CCR1 significantly decreased, while CCR5 increased in LA compared to EA demyelinating regions. Therefore, CCR5 expression within local effector cells such as macrophages and microglia, may reflect the local inflammatory milieu within the lesions. Interestingly, microglia appears to express preferentially other members of the CC chemokine family, including CCL3 and CCL4 [62,65], and various types of injury to the CNS elicit microglial activation [63]. Microglia may display different activity states under different pathological conditions [66]. Microglial activation is generally associated with a change in morphology into an amoeboid appearance with shortened cytoplasmic processes and a rounded cell body accompanied by increased expression of genes involved in immune reactions.

CCR5 is recognized by chemokines CCL3, CCL4 and CCL5. CCR5 seems dispensable for the development of EAE, because CCL3/CCR5 deficient mice have been shown to be fully susceptible to MOG-induced EAE [67]. Such dispensability may support the idea that differential chemokine expression patterns represent differences in disease mechanism that underlie various models of EAE and possibly the distinct patterns of pathology seen in MS [4]. Moreover, in a model for chronic-relapsing EAE, CCR1 and CCR5 blockade with Met-RANTES did not affect leukocyte trafficking despite a modest reduction in disability [68]

The possible role of CCR5 in MS has been further studied in genetic association studies of the human CCR5*Δ32 deletion mutation, that abolishes functional CCR5 on cell surface and may reduce cell entry into lesion sites [69]. Individuals homozygous for the CCR5*Δ32 mutation were found to be resistant to HIV infection [70]. Individuals homozygous for a non functional Δ32 CCR5 develop MS [71] and individuals heterozygous for the Δ32 non-functional CCR5 allele experience prolonged disease free intervals, compared to ones with a fully functional CCR5 receptor [72]. Data has emerged from Finland, suggesting that the lack of CCR5 does not protect from MS, but rather it may predispose to the chronic course of the disease [69]. This would further imply that in view of the redundancy in the chemokine system, CCR5 ligands must be assumed to function through other closely related chemokine receptors [69]. Yet other studies found that the CCR5*Δ32 mutation does not influence susceptibility to MS, neither being protective, nor a risk factor [73-77].

Thus, functional knock-out of CCR5 in humans *per se *confers no protection from MS, and the lack of effect of CCL3 deficiency in mice [67] illustrates redundancy in the chemokine system. Although some of the data on the role of CCR5 in the pathogenesis of MS and EAE appears to be conflicting, the weight of evidence identifies CCR5 as an active participant in the recruitment of inflammatory cells from the circulation, promoting tissue injury in MS and EAE lesions. In this regard, CCR5 expression may be a useful marker to identify effector cells in MS and could be used as a tool for monitoring disease activity [78], and response to treatment [79].

The process of inflammation in EAE is limited at the remission stage of the disease, including substantially reduced numbers of actively phagocytosing macrophages in the CNS. This coincides with diminished expression of CCR1, CCR2 and CCR5 in the CNS. Several non-mutually exclusive scenarios may be postulated to explain the reduced inflammation during the remission stage. One possibility may be that anti-inflammatory chemokine receptors such as CCR3, CCR4, and CCR8, are induced in the CNS. This could occur in combination with a lack of recruitment into the CNS late in the disease due to a decrease in the expression of chemokines and adhesion molecules. Another possibility is the exhaustion of infiltrating leukocytes due to apoptosis. Many studies have demonstrated apoptosis of infiltrating cells in the CNS of animals with EAE [80]. The limitation of inflammation seen in the CNS could also be the result of a diminished antigen-presenting capability.

In conclusion, our findings imply that CC chemokine receptors could all potentially activate and recruit both resident microglia and infiltrating haematogenous cells to sites of CNS inflammation, and provide several potential chemokine receptor targets for therapeutic intervention at different time-points in the disease process, allowing the lessons learned from this model to be applied to human MS. However, it should be remembered that immune cell migration is critically important for active clearance and repair of injured tissues as well as for the delivery of protective immune responses [81-83], a fact that should be closely monitored in future treatment studies in animal models for MS, as well as in clinical trials in humans.

## Conclusion

• Our results demonstrate that the acute and chronic-relapsing phases of MOG-EAE are associated with distinct expression patterns of CCR1, CCR2, and CCR5 mRNA by cells of the macrophage/microglia lineage within the CNS lesions.

• These data support the notion that CCR1, CCR2 and CCR5 mediate recruitment of both infiltrating macrophages and resident microglia to sites of CNS inflammation.

• Detailed knowledge of expression patterns is crucial for the understanding of therapeutic modulation and the validation of CCR1, CCR2 and CCR5 as feasible targets for therapeutic intervention in MS.

## Competing interests

The author(s) declare that they have no competing interests.

## Authors' contributions

Design of studies (DS, SE, EW, TO, HL, A-LB, AE-D), experimental induction of EAE and preparation of tissues (SE, DS, MN), in situ hybridization and immunohistochemistry (DS, SE, MN, AE-D), analysis of data (DS, SE, HL, A-LB, AE-D), writing/reviewing of manuscript (all authors). All authors have read and approved the final manuscript.
